# COVID-19: protocol for observational studies utilizing near real-time electronic Australian general practice data to promote effective care and best-practice policy—a design thinking approach

**DOI:** 10.1186/s12961-021-00772-4

**Published:** 2021-09-07

**Authors:** Andrew Georgiou, Julie Li, Christopher Pearce, Adam McLeod, Nasir Wabe, Rae-Anne Hardie, Guilherme Saffi Franco, Chisato Imai, Gorkem Sezgin, Judith Thomas, Zhaoli Dai, Muhammad Kashif Sheikh, Amanda Proposch, Stephen Weeding, Brendon Wickham, Tony Badrick, Darnel Murgatroyd

**Affiliations:** 1 Australian Institute of Health Innovation, Macquarie University, Macquarie Park, NSW 2109 Australia; 2 Outcome Health, Blackburn, VIC 3130 Australia; 3 Department of General Practice, Monash University, Clayton, VIC 3168 Australia; 4 Gippsland Primary Health Network, Traralgon, VIC 3844 Australia; 5 Eastern Melbourne Primary Health Network, Box Hill, VIC 3128 Australia; 6 South Eastern Melbourne Primary Health Network, 3202, Heatherton, VIC Australia; 7 Royal College of Pathologists of Australasia, Quality Assurance Programs, St Leonards, NSW 2065 Australia; 8 Digital Health Cooperative Research Centre, Sydney, NSW 2006 Australia; 9 Macquarie University, Level 6, 75 Talavera Rd., Macquarie Park, NSW 2109 Australia

**Keywords:** COVID-19, General practice, Design thinking, Quality of care, Observational studies, Pandemics

## Abstract

**Background:**

Health systems around the world have been forced to make choices about how to prioritize care, manage infection control and maintain reserve capacity for future disease outbreaks. Primary healthcare has moved into the front line as COVID-19 testing transitions from hospitals to multiple providers, where tracking testing behaviours can be fragmented and delayed. Pooled general practice data are a valuable resource which can be used to inform population and individual care decision-making. This project aims to examine the feasibility of using near real-time electronic general practice data to promote effective care and best-practice policy.

**Methods:**

The project will utilize a *design thinking* approach involving all collaborators (primary health networks [PHNs], general practices, consumer groups, researchers, and digital health developers, pathology professionals) to enhance the development of meaningful and translational project outcomes. The project will be based on a series of observational studies utilizing near real-time electronic general practice data from a secure and comprehensive digital health platform [POpulation Level Analysis and Reporting (POLAR) general practice data warehouse]. The study will be carried out over 1.5 years (July 2020–December 2021) using data from over 450 general practices within three Victorian PHNs and Gippsland PHN, Eastern Melbourne PHN and South Eastern Melbourne PHN, supplemented by data from consenting general practices from two PHNs in New South Wales, Central and Eastern Sydney PHN and South Western Sydney PHN.

**Discussion:**

The project will be developed using a design thinking approach, leading to the building of a meaningful near real-time COVID-19 geospatial reporting framework and dashboard for decision-makers at community, state and nationwide levels, to identify and monitor emerging trends and the impact of interventions/policy decisions. This will integrate timely evidence about the impact of the COVID-19 pandemic related to its diagnosis and treatment, and its impact across clinical, population and general practice levels.

## Background

Since its identification in December 2019, SARS-CoV-2 and its associated coronavirus disease (COVID-19) has had a devastating effect on communities around the world, leading to a rapidly escalating mortality toll of over a million people [1]. Health systems around the world have been forced to make rapid choices about how to prioritize care, manage infection control and maintain reserve capacity for future disease outbreaks [2]. The interruption of normal patterns of healthcare and the suspension of services has meant that the pandemic has also had a major impact on the detection and treatment of many non-COVID-19 conditions [3].

The healthcare consequences associated with the COVID-19 pandemic range from people’s avoidance of contact with healthcare settings, either for fear of contracting COVID-19 or as a means of reducing pressure on the health system [4], or because of the increased financial stress caused by the pandemic [5]. Early evidence from an Australian Bureau of Statistics Household Impacts of COVID-19 Survey showed that one in 14 Australians (1.4 million adults) were unable to see their general practitioner (GP) or other health professional in person during the early April 2020 to early May 2020 period [6]. Australian Medicare figures revealed that pathology testing fell by almost 30%; consultations with specialists also fell by 8%, and medical operations by 27% [5]. The pandemic has had a disproportionate impact on disadvantaged communities, helping to entrench inequalities, as a consequence of the economic measures used to contain the virus [2]. The slump in general practice visits may have detrimental and long-term effects on patient care and outcomes, especially if it impacts on the diagnosis of new conditions, recommended disease and cancer screening programmes, or ongoing monitoring of patients with chronic disease [7, 8].

Whilst the initial focus has been on COVID-19 and its mortality, the effects of SARS-CoV-2 are evolving into (at this stage) a three-pronged disease:

- Acute COVID-19, the disease responsible for most of the initial morbidity and in particular mortality [9]
- Long COVID, the emerging long-term effects of SARS-CoV-2 infection, with or without initial hospitalization [10, 11]
- Paediatric disease, variously called paediatric inflammatory multisystem syndrome temporally associated with SARS-CoV-2 (PIMS-TS) in Europe and also known as multisystem inflammatory syndrome in children (MIS-C) in the United States) [12].

Whilst general practices were initially severely affected by the drop in visits in the immediate aftermath of the COVID-19 outbreak, the utilization of Medicare funding for telehealth helped to reinforce community access to general practices and non-GP specialists. This change represented a potentially significant shift in how patient care can be delivered in the future. Australian Bureau of Statistics figures over a 4-week period across early April to early May 2020 showed that one in six Australians (17%) used telehealth services (mostly in the form of telephone calls) [6]. Despite the importance of telehealth initiatives, many patients still require a face-to-face option [13]. In recognition of this, on 30 March 2020 the Commonwealth Government announced temporary increases to incentive payments (Practice Incentives Program Quality Improvement) for general practice to increase support for bulk-billed services with the aim of supporting essential face-to-face care access for patients [14].

Identifying the impact of COVID-19 on the healthcare system in near real time is an urgent priority for critical decision-making about resource allocation, control measures, decision-making, policy development and community communication. Primary healthcare has moved into the front line as COVID-19 testing transitions from hospitals to multiple general practice providers, where tracking testing behaviours can be fragmented and delayed.

This paper outlines a project funded by the Digital Health Cooperative Research Centre (DHCRC) which will examine the feasibility of using near real-time electronic general practice data to promote effective care and best-practice policy through the following mechanisms: (1) the building of a near real-time COVID-19 geospatial reporting framework; (2) the generation of timely and critical evidence about the impact of COVID-19; (3) the building of a predictive geospatial analytics dashboard for timely, evidence-based decision-making; and (4) the establishment of an evidence-based suite of general practice outcome measures required to monitor the quality and effectiveness of care related to incidence and prevalence, recovery and mortality.

The project was formally identified initially as a project concept proposal to the DHCRC involving a collaboration between Macquarie University, Outcome Health, Gippsland, Eastern Melbourne, South Eastern Melbourne Primary Health Networks (PHNs) and the Royal College of Pathologists of Australasia Quality Assurance Programs. The proposal was assessed by the DHCRC using its eligibility threshold criteria to meet its research and investment framework. Following this, we developed a project plan which was reviewed by the DHCRC according to its merit and value criteria, which led to the establishment of a project agreement (17 June 2020).

## Methods

### Design thinking

The codesign aspects of the project will involve all the project collaborators (PHNs, general practices, consumer groups, researchers, and digital health developers, pathology professionals) as part of an integrated collaboration to enhance the development of meaningful and translational project outcomes. The project will utilize a *design thinking* approach to incorporate user needs and feedback throughout the development of the research process. This approach involves rounds of ideation, prototyping and testing [15] to enhance understanding of underlying problems or unpredictable situations [16].

The project builds upon an established collaboration between Macquarie University, the Royal College of Pathologists Quality Assurance Programs, Outcome Health and Gippsland, Eastern and South Eastern PHNs, which evaluated the appropriateness and quality use of pathology in general practice [17] using pooled electronic general practice data [18]. This project provided an overview of pathology testing within Australian general practice, along with reporting on variation across key indicators including vitamin D testing, glycated haemoglobin (HbA1c) and kidney function tests for monitoring type 2 diabetes, ferritin testing for iron deficiency and prostate-specific antigen (PSA) testing [19].

### Population studies

A series of population studies will be carried out over the period July 2020–December 2021 utilizing near real-time electronic general practice data to promote effective care and best-practice policy. The type of studies examining the impact of the COVID-19 pandemic will include (1) the uptake of general practice telehealth services; (2) medication prescribing; (3) the impact on pathology testing; and (4) general practice consultations to long-term aged care facilities.

Data examination and analysis will be performed using Stata/MP 16 (StataCorp) [20], R v4.0.2 (R Core Team) [21] and SAS 9.4 (SAS Institute) statistical software [22]. Statistical methods will include descriptive and inferential statistics, depending on the components of the project. Where spatial or temporal evaluations are required, stochastic (e.g., mixed models) and deterministic models (e.g., Bayesian structural time series) will be developed as per the study aims, and if applicable, incorporated into machine learning for building prediction models. The methods will be structured according to the reporting of studies conducted using observational routinely collected health data or pharmacoepidemiology data (RECORD-PE) [23]. The RECORD-PE checklist deals specifically with real-world research and evaluation using data from electronic health records such as that provided by electronic general practice sources [23].

The studies will be centred on 450 general practices within three Victorian PHNs, Gippsland PHN, Eastern Melbourne PHN and South Eastern Melbourne PHN. These PHNs cover metropolitan and rural regions across a combined area totalling 48,903 km^2^, delivering healthcare to 3,132,382 Australians [19]. Data will be supplemented by participation of two PHNs from New South Wales, Central and Eastern Sydney PHN and South Western Sydney PHN, with Central and Eastern Sydney PHN incorporating a further 350 general practices.

### Data source

Outcome Health, as a data custodian, uses its POpulation Level Analysis and Reporting (POLAR) Data Space to provide a secure and comprehensive digital health platform which collects data from consenting general practices across participating PHNs [17, 18, 24–26]. Data variables include de-identified demographic information about patients and general practices, as well as visit records (diagnosis, past history, medications, immunization, radiology) and pathology test records (test name and result).

### Sample size considerations

The research partners in this project have been amongst the first in Australia to successfully use and analyse electronic patient data from general practice and evaluate patient outcomes in both cross-sectional and longitudinal studies [18, 19]. This body of evidence (initially based on > 350 practices) includes a comprehensive assessment of outcomes for patients with type 2 diabetes [18], and an investigation of iron deficiency diagnosis in general practice [27]. The research outlined in this paper is centred on data from > 800 general practices, representing a total of > 4 million patients (~ 56% female). This sample will provide sufficient scope to detect significant variation in practices across patient and general practice demographic domains.

### Project components

The study will involve the building of a meaningful near real-time COVID-19 geospatial reporting framework and dashboard for decision-makers at community, state and nationwide levels, to identify and monitor emerging trends and the impact of interventions/policy decisions. This will include:

1. The generation of timely and critical evidence (e.g., publicly available reports, peer-reviewed publications and media presentations) about the impact of COVID-19 across the following care level dimensions:
  *Clinical:* Patient-level impacts based on:
    *Diagnostics* (most common COVID-19 symptoms; alarm flags for COVID-19, e.g. white blood cells, lymphocytes, platelet counts; risk factors, e.g. smoking, respiratory failure, etc.);
    *Medications* (the dangers of prescribing antiviral drugs, e.g. drug–drug, drug–disease interactions; risks associated with chloroquine/hydroxychloroquine, angiotensin-converting enzyme [ACE] inhibitors, aggravation of symptoms, etc.); and
    *Patient care* (Considerations during pregnancy, care of infants, use of nebulisers, recommendations for paediatric patients etc.).
  *Population:* What populations are being impacted, not only from a direct COVID-19 perspective, but from a regular care perspective (e.g., impact on chronic disease, preventative care and mental health).
  *Business:* What impact has COVID-19 had on general practice (e.g., number and types of services, tests, medications, etc.) from a business and financial perspective? Types of interaction with patients, telehealth vs face-to-face, how has this worked, what are the immediate and future implications? How has this impacted patient care in relation to population and clinical care?
2. Development of a predictive geospatial analytics dashboard for timely, evidence-based decision-making at community, state and nationwide levels to include:
  Visual representations of patient status and clinical environments that change dynamically in near real-time.
  Risk prediction tools based on statistical modelling incorporating demographics, comorbidities, patient symptoms and risk factors.
  Incorporation of displays and decision support features tailored to the needs and preferences of key recipients including GPs, PHNs and government health agencies.

### Patient and public involvement in research

Each of the project partners have strong records of collaborative research which includes engagement with patients and the public. The Macquarie University research team has an established Consumer Reference Group made up of consumer representatives who have contributed to the design, development and promulgation of research aims [28, 29]. The Consumer Reference Group was initiated following a national stakeholder forum (which brought together representatives from 14 stakeholder groups including patient organizations, clinicians and healthcare professionals) that outlined key patient safety challenges related to the utilization of digital health and the diagnostic process [29]. The project will draw on established PHN stakeholder (patient and public) involvement using design thinking approaches to inform research questions, the design and conduct of studies and choice of outcome measures.

### Limitations

The electronic general practice data available for this project represent a rich source of information about general practice activity and patient care processes. However, there are limitations in the data, including variables that may not be recorded in a standardized way, or inconsistencies in how information is documented and identified. In some cases this may be due to the lack of comparability between software packages used by general practices, specialists, pathology and medical imaging providers. Furthermore, free-text data of comorbidities and diagnoses increase the possibility of underreporting. Nevertheless, the collection of general practice data directly from general practices at the time of patient care provides a valuable source of pooled data, which can help to power major research investigations.

### Research governance

The project will establish an operational and governance structure consisting of the following:

a Project management team (PMT) which will consist of representatives of the collaborating partners (Macquarie University, Outcome Health, Gippsland, Eastern Melbourne, and South Eastern Melbourne PHNs). The PMT will be responsible for the day-to-day functioning of the Project. It will draw on a team of clinical, technical and digital health analytics experts drawn from each of the collaborating partners, who will provide regular timely advice and guidance to generate meaningful robust, validated outcomes. This will include liaison with all stakeholder groups (general practice, nurses, managers and consumers).
b Project Control Group that will consist of key representatives from the collaborating partners and the DHCRC with responsibility to oversee the Project’s progress, priorities and direction. The Project Control Group will meet quarterly and be presented with interim reports for consideration during the course of the Project.

### Ethics and dissemination

Outcome Health has received ethical approval from the Royal Australian College of General Practitioners National Research and Evaluation Ethics Committee (NREEC) 17-008 POLAR GP data warehouse. The Macquarie University Human Research Ethics Committee (HREC) Medical Sciences Committee has granted approval to the project (Reference no. 5202067517176).

The operational objectives of the project have been designed to contribute significantly to the enhancement of quality general practice care in response to COVID-19. Findings will be regularly communicated to GPs, PHNs, the community and nationally through near real-time snapshot reports. Findings will be disseminated in peer-reviewed academic journals and presentations (national and international conferences and industry forums).

## Discussion

General practice services have a critical role to play in Australia’s response to COVID-19, not least because they are often the place of initial health system contact for most Australians [30]. They are essential as suppliers of information to the community in terms of healthcare advice to mitigate the spread of disease and protect the health of the community [31].

General practice activity is also key to identifying and monitoring the health of the community, providing an early warning system of the spread of the pandemic and targeting areas where healthcare may be avoided or delayed, leading to the possibility of missed diagnoses, medications and treatment, which may have serious future consequences for patients and the healthcare system [5].

Until recently, research into Australian general practice has been hampered by the lack of reliable data and expertise in data management, information systems and quality improvement infrastructures [32]. Pooled general practice data are a valuable resource, which can be used to inform decision-making including individual care, population health and business and government strategy. The research partners in this project have been amongst the first in Australia to successfully use and analyse electronic patient data from general practice and evaluate patient outcomes in both cross-sectional and longitudinal studies [17, 19].

The operational objectives of this project have been designed to contribute to the enhancement of quality general practice in response to the COVID-19 pandemic, providing a foundation for improved care monitoring and planning and more timely decision-making and evidence-based policy development.

## Data Availability

Not applicable.

## Abbreviations

PHNs: Primary health networks
GP: General practitioner
PIMS-TS: Paediatric inflammatory multisystem syndrome
MIS-C: Multisystem inflammatory syndrome in children
DHCRC: Digital Health Cooperative Research Centre
HbA1c: Glycated haemoglobin
PSA: Prostate-specific antigen
POLAR: POpulation Level Analysis and Reporting
RECORD: Reporting of studies conducted using observational routinely collected data
PMT: Project management team
NREEC: National Research and Evaluation Ethics Committee
HREC: Human Research Ethics Committee
